# Twelve-month outcomes of a community-based, father-daughter physical activity program delivered by trained facilitators

**DOI:** 10.1186/s12966-024-01648-w

**Published:** 2024-09-11

**Authors:** Lee M. Ashton, Anna T. Rayward, Emma R. Pollock, Stevie-Lee Kennedy, Myles D. Young, Narelle Eather, Alyce T. Barnes, Daniel R. Lee, Philip J. Morgan

**Affiliations:** 1 Centre for Active Living and Learning, College of Human and Social Futures, School of Education, University of Newcastle, University Drive, Awabakal Country, Callaghan, NSW 2308 Australia; 2 Active Living and Learning Research Program, Hunter Medical Research Institute (HMRI), Lot 1 Kookaburra Circuit, New Lambton Heights, NSW 2305 Australia; 3 School of Medicine and Public Health, The University of Newcastle, Newcastle, NSW Australia; 4 The National Centre of Implementation Science (NCOIS), The University of Newcastle, Newcastle, NSW Australia; 5 Population Health Research Program, Hunter Medical Research Institute, New Lambton Heights, NSW Australia; 6 Hunter New England Population Health, Hunter New England Local Health District, Newcastle, NSW Australia; 7 College of Engineering, Science and Environment, School of Psychology, University of Newcastle, Callaghan, NSW Australia

**Keywords:** Fathers, Girls, Community, Physical activity, Parenting

## Abstract

**Background:**

Dads and Daughters Exercising and Empowered (DADEE) is a program targeting fathers/father-figures to improve their daughters’ physical activity and well-being. Previous randomised controlled efficacy and effectiveness trials of *DADEE* demonstrated meaningful improvements in a range of holistic outcomes for both fathers and daughters in the short-term. This study aims to assess the long-term impact (12-months) of the program when delivered in the community by trained facilitators.

**Methods:**

Fathers/father-figures and their primary school-aged daughters were recruited from Newcastle, Australia into a single-arm, non-randomised, pre-post study with assessments at baseline, 10-weeks (post-intervention) and 12-months. The 9-session program included weekly 90-min educational and practical sessions, plus home-based tasks. The primary outcome was fathers’ and daughters’ days per week meeting national physical activity recommendations (≥ 30 min/day of MVPA for fathers, ≥ 60 min/day MVPA for daughters). Secondary outcomes included physical activity, screen time, self-esteem, father-daughter relationship, social-emotional well-being, parenting measures, and process outcomes (including recruitment, attendance, retention and program acceptability).

**Results:**

Twelve programs were delivered with 257 fathers (40.0 ± 9.2 years) and 285 daughters (7.7 ± 1.9 years). Mixed effects regression models revealed significant intervention effects for the primary outcome, with fathers increasing the days/week meeting physical activity recommendations by 27% at 10-weeks (*p* < 0.001) and by 19% at 12-months (*p* < 0.001) compared with baseline. Likewise, for daughters there was a significant increase by 25% at 10-weeks (*p* < 0.001) and by 14% at 12-months (*p* = 0.02) when compared to baseline. After conducting a sensitivity analysis with participants unaffected by COVID-19 lockdowns (*n* = 175 fathers, *n* = 192 daughters), the primary outcome results strengthened at both time-points for fathers and at 12-months for daughters. Additionally, the sensitivity analysis revealed significant intervention effects at post-program and 12-months for all secondary outcomes in both fathers and daughters. Furthermore, the process outcomes for recruitment capability, attendance, retention and satisfaction levels were high.

**Conclusions:**

Findings provide support for a sustained effect of the DADEE program while delivered in a community setting by trained facilitators. Further investigation is required to identify optimised implementation processes and contextual factors to deliver the program at scale.

**Trial registration:**

ACTRN12617001450303. Date registered: 12/10/2017.

**Supplementary Information:**

The online version contains supplementary material available at 10.1186/s12966-024-01648-w.

## Background

There are growing concerns about the disease burden of physical inactivity in the Australian population [1]. Two key demographics of concern are girls and men, with 91% of girls aged 11–17 [2] and 68% of adult men [3] not meeting physical activity guidelines. There are numerous sociocultural factors contributing to girls being less active than boys at all ages [4]. For example, girls face cultural biases that limit their opportunities to participate in physical activity and sport [5] and are consequently far less likely to develop the necessary skills for lifelong participation [6]. For men, fatherhood is associated with decreased physical activity levels [7]. Targeting physical activity levels in fathers has been identified as a way of improving their well-being and the well-being of their children [8]; however, fathers are far less likely to be involved in family-based physical activity interventions than mothers [9]. Fathers are also less likely to participate in co-physical activity with their daughters than their sons [10].

Dads and Daughters Exercising and Empowered (DADEE) was developed as a novel approach that brings fathers/father-figures into their daughters’ lives through physical activity, empowering both with the knowledge and skills to combat gender bias. In an efficacy randomised controlled trial (RCT) that was delivered by the research team, fathers and daughters who participated in *DADEE* achieved a range of improved health behaviours, including increased physical activity levels and reduced screen time. Fathers’ physical activity parenting practices also improved, alongside family relationship and daughters’ fundamental movement skill (FMS) competency and social-emotional well-being [11–13]. In a community-based effectiveness RCT, most of these improvements were again observed, with very high program attendance, satisfaction and fidelity [13, 14]. However, these promising results were assessed in the short-term only, which is a common limitation of many family-based, physical activity programs [15]. In the field, many studies have contributed evidence towards program development and intervention testing (i.e., efficacy and effectiveness trials) with few focusing on translational research goals of long-term intervention replication and dissemination [16].

This present study aimed to progress the evidence base by testing whether the positive outcomes for fathers and daughters established in the previous *DADEE* RCTs could be sustained long-term (12-months) when delivered in the community by trained facilitators.

## Methods

### Study design

The study employed a non-randomised, pre-post trial design, with evaluations conducted at baseline, 10-weeks (post-program), and 12 months. Data for this study were gathered from 12 programs (four programs each year between 2017–2019), led by trained facilitators in community settings (three local primary schools after school hours). The study was registered in advance with the Australian New Zealand Clinical Trials Registry (ACTRN12617001450303) and institutional ethics approval (H-2014–0330) was obtained for the research.

### Participants

Between 2017–2019, participants were recruited from Newcastle, New South Wales, Australia. We used a variety of recruitment strategies including a university media release highlighted in local news outlets (television news, newspaper, and radio), social media outreach (via Facebook and X—formerly Twitter) and school newsletter advertisements. Fathers were eligible if they were ≤ 65 years of age and were a father or a father-figure (such as a grandfather, uncle, stepfather, or male role model) to a primary-school aged daughter (i.e., aged 4–12 years). Throughout this paper, the term "father" encompasses both biological fathers and father figures. Fathers who had previously participated in the program (prior to 2017) were eligible if they enrolled with a daughter who had not previously participated. Before enrolment, fathers who were unable to attend the entire program were excluded, while fathers with significant pre-existing health conditions (e.g., cardiovascular disease or recent chest pains during exercise) required a doctor's clearance. Daughters were eligible to participate if they were currently attending primary school from kindergarten to Year 6. Daughters with pre-existing medical conditions that may affect their ability to participate in physical activity were required to obtain a doctor’s clearance. Fathers could enrol with one or more daughters, with a maximum limit of three daughters allowed. For fathers enrolling with multiple daughters, the questionnaires were completed in relation to their eldest daughter. All fathers provided written informed consent, along with child assent.

### The ‘Dads and Daughters Exercising and Empowered (DADEE)’ Intervention

Information regarding the theoretical underpinnings, structure and weekly session content can be found in the previous *DADEE* effectiveness RCT [14]. In summary, *DADEE* was informed by extensive formative work [17–20] and based upon key constructs of Self-Determination Theory (i.e., autonomy, competence, relatedness) [21] and Social Cognitive Theory (e.g., self-efficacy, goals, social support) [22]. Constructs of *relatedness* (i.e., desire to connect and care for others) and *social support* were integrated into the program by promoting ‘reciprocal reinforcement’, where fathers were encouraged to role model positive behaviours and promote physical activity for their daughters, and vice versa. To enhance *autonomy* (i.e., choice and control), participants were given multiple options for completing program activities and home tasks. Also, the program encouraged fathers and daughters to choose challenges that led to success, regardless of age, fitness, or skill level. These variations aimed to enhance participants’ *perceived competence* and *self-efficacy* for physical activity. Additional strategies such as verbal persuasion and role modelling were utilised to promote *self-efficacy*. To enhance positive *outcome expectations*, participants received information about the physical, mental, social, and emotional benefits of co-physical activity. Additionally, they learned engaging ‘active’ games designed to be done together that were both fun and optimally challenging. Fathers and daughters were also encouraged to set personal and family-based physical activity *goals* and track their progress. See Additional File 1 for TIDieR checklist for intervention description. All sessions during the 9-week program were held as a group with a limit of 25 families per program. The programs were held at a local primary school which had suitable facilities to undertake education and practical components. Each session included three components, as detailed below:

(i) *A 15-min education session with fathers and daughters together;* each session started with fathers and daughters working through key social-emotional constructs (e.g., self-control, persistence, resilience, kindness, bravery, positivity, critical thinking, self-reliance). This included discussion activities and tips on how to apply to their everyday lives (i.e., school, work, relationships, and sporting/ extra-curricular activities). In addition, fathers and daughters were provided with critical thinking skills to identify, navigate and challenge gender prejudice that infiltrates all aspects of girls’ lives, particularly in sport and physical activity.
(ii) *A 30-min education session for fathers and daughters conducted separately;* Each week there was a key education session focus (i.e., physical activity, female role models, sport skills, screen time) for both fathers and daughters. For fathers, the education sessions also focused on proven parenting strategies to improve their daughters’ social-emotional well-being, sports skills and physical activity levels. While the daughters’ education sessions provided further information on developing key social-emotional skills. Program educational content was kept simple, and older girls were paired with younger girls to assist them during education session activities.
(iii) *a 45-min practical session where fathers and daughters participate together;* each session focused on three key areas: rough and tumble play, FMS practise (e.g., kick, catch, strike, bounce, overhand throw and underhand throw) and fitness activities. Facilitators described variations of different activities and fathers were trained to adapt activities appropriately for age and skill levels.
(iv) To enhance family support, mothers/partners and siblings were invited to attend one of the sessions (session four), where all family members participated in the activities together. Each week, participants were provided with home-resources to practise and improve confidence in the sport skills and reinforce what was learnt in each session, this included:
*A Father’s Logbook*—to document brief home-based tasks including; setting SMART goals, tracking physical activity and co-activity with daughter and completing weekly ‘dad tasks’ (e.g., ‘*instigate an active backyard game with your daughter’*).
*A Sport Skills Booklet*—containing key coaching points and engaging practise activities relating to the six FMS, that were uniquely designed for fathers and daughters.
*A Daughters’ Booklet—*containing tasks relating to development of social-emotional skills, co-physical activity promotion to nurture the father–daughter relationship and instructions for using the mobile *DADEE* App.
*A mobile App –* containing a variety of fun physical activities for daughters and fathers to complete and track together weekly (e.g., sock wrestle). This was only used in four programs delivered in 2017 and was removed for the remaining eight trials in 2018 and 2019 due to financial restraints of maintaining and updating the app. For 2018 and 2019 programs, all the activities were moved to print format in the *Daughter’s booklet.*

### Facilitator recruitment and training

A focused recruitment strategy was employed to enlist *DADEE* facilitators, with an emphasis on targeting individuals with previous experience working with children, adept communication with families, and the ability to conduct practical sessions safely and efficiently. Across the three years, *n* = 13 physical education teachers from local schools and *n* = 13 Bachelor of Education students from the University of Newcastle were recruited via email. All *DADEE* programs were delivered by an experienced physical education teacher, with some programs also involving secondary/apprentice facilitators who were current Bachelor of Education students. Facilitators participated in a three-day, in-person training workshop conducted by the lead researcher (PJM) at the University of Newcastle. The workshop centred on *DADEE* program details (i.e., rationale, structure, and background), and guidance for delivering informative and engaging educational and practical sessions for fathers and their daughters. In addition, facilitators were equipped with resources to aid program facilitation. These resources included PowerPoint slides covering all educational session content, including presentation notes detailing how to deliver each slide, videos showcasing practical activities, and a practical handbook offering information about setting up, delivering, and modifying practical activities.

### Outcomes

For the 12 *DADEE* programs, assessments at baseline, 10-weeks (post-program), and 12 months occurred between October 2017 to October 2020. To note: all programs were completed by December 2019 and were not affected by the Covid-19 pandemic, however for participants that did the program in 2019, the 12-month measures were collected after the NSW Government declared a strict lockdown in 2020. A full description of all outcome measures is provided in Table 1. Demographic information included: participant age, fathers’ employment status, Aboriginal or Torres Strait Islander identity, education level, annual household income, relationship status, employment status and country of birth. Socioeconomic status was determined using the Australian postal area index of relative socioeconomic advantage and disadvantage [23]. All self-reported data were collected and managed using SurveyMonkey (Survey Monkey Inc, San Mateo, California, USA) for 2017 programs and REDCap electronic data capture tools for 2018 and 2019 programs [24, 25]. Both online survey platforms were hosted at University of Newcastle.

**Table 1:** Overview of primary and secondary outcome measures

Measure	Description	Timepoint
***Primary outcome***		
* Fathers’ MVPA (self-reported days/week meeting physical activity recommendations- which is* ≥ *30 min/day of MVPA)*	**• Measurement tool:** A single item question from the Australian Bureau of Statistics *'Australian Health Survey'* [26] **• Metrics/questions:** Single item question: *“On how many of the past 7 days did you engage in a total of 30 min or more of physical activity, that was hard enough to raise your breathing rate? This may include brisk walking, sport, exercise or cycling for recreation or to get to and from places. It should not include housework or physical activity that may be part of your job.”* **• Completed by:** Fathers.	Baseline, 10-weeks, 12-months
* Daughters’ MVPA (self-reported days/weekmeeting physical activity recommendations- which is* ≥ *60 min/day of MVPA))*	**• Measurement tool:** A single item question from the Australian Bureau of Statistics *'Australian Health Survey'* [26]. **• Metrics/questions:** Single item question: *“On how many of the past 7 days did your daughter/s engage in sport, physical activity or active play for a total of at least 60 min? Some examples include playing soccer, netball, basketball, rugby league or union, Australian Rules football, swimming, walking or riding a bicycle to or from school, skipping, running, rollerblading, dancing or any activity that made your daughter/s huff and puff.”* **• Completed by:** Father proxy.	Baseline, 10-weeks, 12-months
***Secondary outcomes***		
* Father’s self report MVPA (mins/week)*	**• Measurement tool:** Adapted version of the Godin Leisure Time Exercise Questionnaire [27]. **• Metrics/questions:** Fathers reported average weekly bouts of moderate and vigorous physical activity and average bout length. Values in each category were multiplied and summed to give an overall measure of weekly MVPA. **• Completed by:** Fathers.	Baseline, 10-weeks, 12-months
* Father-child co-physical activity (days/week)*	**• Measurement tool:** 2-items adapted from the Youth Media Campaign Longitudinal Survey [28]. **• Metrics/questions:** Fathers reported on days per week they were physically active with their child one-on-one and with one or more family member. **• Completed by:** Fathers.	Baseline, 10-weeks, 12-months
* Father’s and daughter’s screen time*	**• Measurement tool:** Adapted version of the Adolescent Sedentary Activity Questionnaire [29]. **• Metrics/questions:** Fathers reported the total time they spent sitting using screens (of any kind) for anything outside of work on each day in the previous week. Fathers also answered these questions on behalf of their daughters. **• Completed by:** Fathers.	Baseline, 10-weeks, 12-months
* Father involvement*	**• Measurement tool:** Using selected subscales from the validated *Inventory of Father Involvement* [30]. **• Metrics/questions:** Each subscale score was created by asking fathers to report, on a 7-point Likert scale (1 = very poor, 7 = excellent), on “how good a job” they were doing on indicators of father involvement (mother support, praise and affection, time and talking together, attentiveness) and taking the mean. Scale range is 1 to 7 for each sub-scale. **• Internal consistency on current sample:** Mother support: α = 0.80, praise and affection α = 0.85, time and talking together α = 0.90, attentiveness: α = 0.77. **• Completed by:** Fathers.	Baseline, 10-weeks, 12-months
* Daughters’ self-esteem*	**• Measurement tool:** Using self-esteem subscale from the validated Kindl-R questionnaire [31, 32] **• Metrics/questions:** The subscale score was created by asking fathers to report on 4 items relating to daughter’s esteem (e.g., *was proud of herself, felt on top of the world, was pleased with herself and had lots of good ideas*) using a 5-point Likert scale (1 = never, 5 = all the time), and taking the mean. After transformation, the instrument delivers values from 0 to 100 with higher values indicating higher self-esteem. **• Internal consistency on current sample:** α = 0.78 **• Completed by:** Father-proxy.	Baseline, 10-weeks, 12-months
* Family functioning*	**• Measurement tool:** Using family subscale from the validated Kindl-R questionnaire [31, 32]. **• Metrics/questions:** Subscale score was created by asking fathers to report on 4 items relating to family functioning (e.g., *daughter gets on well with parents, daughters felt fine at home, we argued at home, daughter felt I was bossing her around),* using a 5-point Likert scale (1 = never, 5 = all the time), and taking the mean. After transformation, the instrument delivers values from 0 to 100 with higher values indicating higher family functioning. **• Internal consistency on current sample:** α = 0.80 **• Completed by:** Father proxy.	Baseline, 10-weeks, 12-months
* Daughters’ social-emotional well-being*	**• Measurement tool:** Devereux Students Strengths Assessment (DESSA) validated in parents with children in kindergarten through to eighth grade [33]. **• Metrics/questions:** The DESSA is a 72-item questionnaire organised into 8 social-emotional competency scales: self-awareness (7 items); social-awareness (9 items); Self-management (11 items); goal-directed behaviour (10 items); relationship skills (10 items); personal responsibility (10 items); decision making (8 items); optimistic thinking (7 items). Each item is scored on a 5-point Likert scale (0 = Never, 4 = Very Frequently). After transformation, each sub-scale can be categorised 28–40 = Need for instruction; 41–59 = Typical; 60–72 = Strength) Social-emotional composite score is obtained by adding all 8 sub-scale scores. **• Internal consistency on current sample:** total composite; α = 0.97, self-awareness; α = 0.78, social-awareness; α = 0.84, Self-management; α = 0.83, goal-directed behaviour; α = 0.87, relationship skills; α = 0.88, personal responsibility; α = 0.83, decision making; α = 0.84, optimistic thinking; α = 0.82. **• Completed by:** Father proxy.	Baseline, 10-weeks, 12-months
* Father-daughter relationship*	**• Measurement tool:** The disciplinary warmth and personal relationships subscales from the *Parent Child Relationships Questionnaire* which has been validated in school-age children [34]. **• Metrics/questions:** Fathers were asked 14 questions (response options 1 = Hardly at all to 5 = Extremely much) which were used to create 7 interim sub-scores. The mean of 3 sub-scores were used to create the disciplinary warmth subscale. The mean of 4 sub-scores were used to create personal relationships subscale. Scale range is 1 to 5 for each sub-scale. **• Internal consistency on current sample:** α = 0.89 **• Completed by:** Fathers.	Baseline, 10-weeks, 12-months
*Process outcome measures*		
* Recruitment capability*	**• Measurement tool:** Audit of study enrolment logs. **• Indicator of success** ^**a**^ **:** Achievement of recruitment targets for participants (recruitment of 240 families to 12 *DADEE* programs across three years).	Baseline
* Attendance*	**• Measurement tool**: Assessed using workshop attendance checklists at the 9-weekly sessions. **• Metrics/questions**: Reported as % attendance on average across the nine weeks. **• Completed by:** Program facilitators **• Indicator of success:** at least 70% average attendance on average across the nine weeks.	Post-program (10-weeks)
* Retention rate*	**• Measurement tool:** Audit of post-programme assessment logs and assessed using the proportion completing all post-program assessments. **• Indicator of success:** A benchmark of ≥ 85% of daughters and dads retained at post-programme assessments.	Post-program (10-weeks)
* Program satisfaction*	**• Measurement tool:** Assessed using post-program process evaluation online survey developed for the purpose of the study. **• Metrics/questions:** Questions focused on participants’ satisfaction with overall program and program facilitators (e.g., *Overall, I enjoyed the Dads and Daughters Exercising and Empowered program?*). Responses were on a 5-point Likert scale where strongly disagree = 1 and strongly agree = 5. **• Completed by:** Fathers **• Indicator of success:** Defined as a mean score of at least 4 out of 5 for satisfaction items measured via questionnaire using a 5-point Likert scale	Post-program (10-weeks)

^a^For replicability purposes, indicators of success were established based on previous process results of the *DADEE* program when delivered in the community [14]

### Data analysis

Baseline participant characteristics were described using frequency distributions for categorical variables and summary statistics for continuous variables stratified by year of program participation. Mixed effects regression models were used to investigate changes in outcomes from baseline to 10-weeks (post-program), and baseline to 12 months. All models were adjusted for SES (SEIFA quintile), cohort year, previous involvement in the program (*n* = 33, 12.8% of fathers had taken part in the program prior to 2017), number of daughters enrolled in program, age of father (father outcomes only) and age of eldest daughter. Poisson regression was used for days/week meeting physical activity recommendations for both fathers and daughters (primary outcome) and co-physical activity (daughter only). Mixed effects linear regression was used for MVPA, screen time, (for both father and daughter) and social emotional well-being (daughters only). Mixed effects negative binomial regression was used for co-physical activity days/week (with daughter and family), while ordinal regression was used for father involvement scores, father-daughter relationship and daughters’ self-esteem. Random individual-level intercepts were included in all models to account for repeated measurements on the same individual. In addition, as multiple daughters could participate from one family, analyses also included a random intercept for family to account for clustering at this level. Model estimates (Incidence rate ratio [IRR], Odds ratio [OR] or average difference) with 95% confidence intervals (CIs) and p-values are presented. For all models, statistical significance is assessed at the 5% level. Missing data were handled using maximum likelihood estimations from the mixed modelling framework [35]. For participants that did the program in 2019, the 12-month measures were collected after the NSW Government declared a strict lockdown in 2020. Therefore, sensitivity analysis was carried out with the regression models described above repeated with the COVID-19 impacted cohorts (2019 programs) removed. Descriptive analyses (i.e., percentage and frequency counts) were conducted to assess the process outcomes including recruitment, retention, attendance and program satisfaction. All statistical analyses were programmed using STATA v17.0 (StataCorp, Texas, USA).

## Results

### Participant flow

**Fig. 1:**
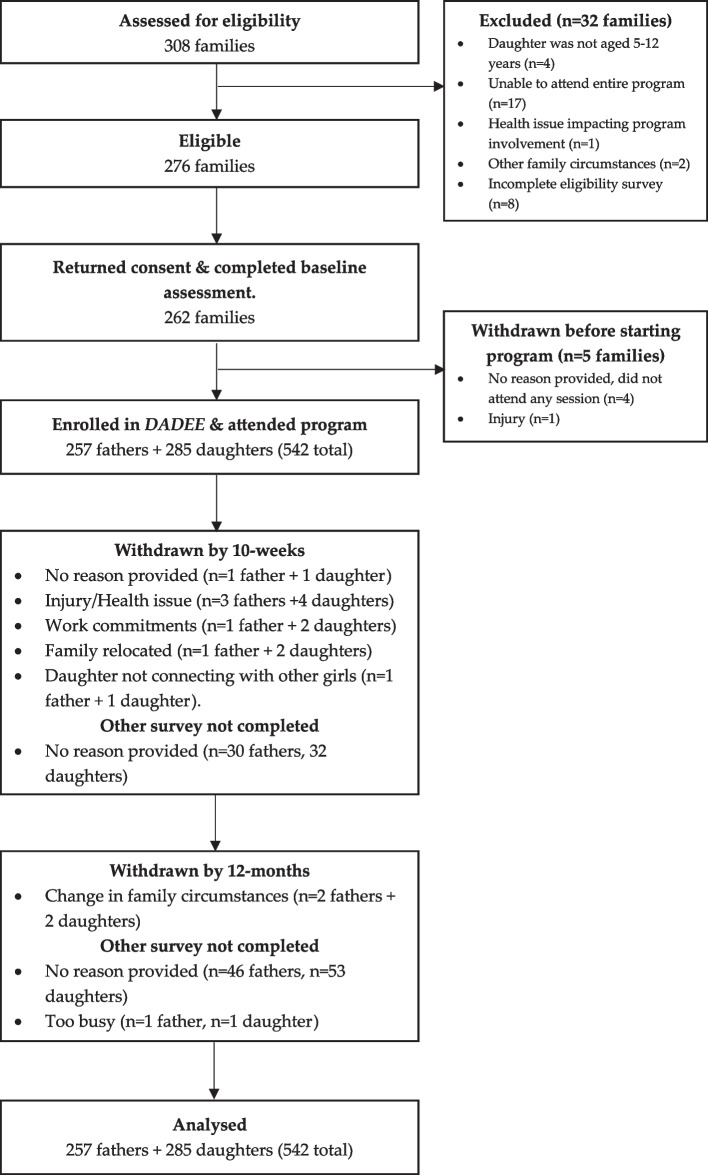
Participant flow through *DADEE* program from 2017–2019

Between 2017–2019, a total of 308 families expressed interest in the *DADEE* program. Of these, 89.6% (*n* = 276 families) met the eligibility criteria (Fig. 1). Most of these were recruited via word of mouth through a friend or family member (63.3%, *n* = 195). Of those eligible, 262 families provided consent and completed baseline assessments. From this, 257 families (257 fathers + 285 daughters) were enrolled in the program and attended at least one session. Most fathers enrolled one daughter (*n* = 230, 89.5%), while *n* = 26 (10.1%) enrolled two daughters and one father (0.4%) enrolled three daughters. Also, *n* = 33 (13%) of fathers had previously participated in the program prior to 2017. A total of 86% (*n* = 220 families) were retained at the end of the program and undertook post-program assessments, while 78% (*n* = 201 families) were retained at 12-months follow-up and undertook post-program assessments.

### Demographics and baseline characteristics

At baseline, the mean age of fathers and daughters was 40.0 ± 9.2 and 7.7 ± 1.9 years. Most fathers were living in areas of medium socio-economic status (*n* = 166, 65%), born in Australia (87.2%), had a university degree or higher university degree (70.4%), working in full-time employment (90.3%) and married (82.9%). Only three (1.2%) fathers were of Aboriginal origin, and no fathers were of Torres Strait Islander origin. Demographic characteristics of study participants split by year of enrolment and the total sample are shown in Table 2. At baseline, the number of days/week that fathers and daughters met physical activity recommendations was 2.6 ± 1.8 and 2.7 ± 1.6. Additional details on the secondary outcomes for physical activity, screentime, parenting outcomes, daughters’ self-esteem, father-daughter relationship and daughters’ social-emotional well-being, stratified by timepoint are shown in Supplementary file 1, Tables 1and 2 (Additional file 2).

**Table 2:** Demographic characteristics of the sample (*n* = 542)

		***Program Year***			
***Variable***	***Category***	***2017 (n*** ** = ** ***79 fathers, n*** ** = ** ***89 daughters)***	***2018 (n*** ** = ** ***96 fathers, n*** ** = ** ***103 daughters)***	***2019 (n*** ** = ** ***82 fathers, n*** ** = ** ***93 daughters***	***Total Sample*** ***(n*** ** = ** ***257 fathers, n*** ** = ** ***285 daughters)***
Age of father at baseline (years)	mean (SD)	42.1 (5.4)	38.6 (10.1)	39.7 (10.6)	40.0 (9.2)
Age of child at baseline	mean (SD)	7.8 (1.7)	7.5 (1.8)	7.9 (2.0)	7.7 (1.9)
SEIFA decile category^a^	Low (1–3)	1 (1.3%)	5 (5.2%)	2 (2.4%)	8 (3.1%)
	Medium (4–7)	50 (63.3%)	65 (67.7%)	51 (62.2%)	166 (64.6%)
	High (8–10)	28 (35.4%)	26 (27.1%)	29 (35.4%)	83 (32.3%)
Aboriginal or Torres Strait Islander status of father	Not indigenous	78 (98.7%)	95 (99.0%)	81 (98.8%)	254 (98.8%)
	Aboriginal Australian	1 (1.3%)	1 (1.0%)	1 (1.2%)	3 (1.2%)
	Torres Strait Islander	0 (0%)	0 (0%)	0 (0%)	0 (0%)
Australian born	No	13 (16.5%)	12 (12.5%)	8 (9.8%)	33 (12.8%)
	Yes	66 (83.5%)	84 (87.5%)	74 (90.2%)	224 (87.2%)
Relationship status	Single	2 (2.5%)	1 (1.0%)	0 (0%)	3 (1.2%)
	In a relationship	1 (1.3%)	1 (1.0%)	2 (2.4%)	4 (1.6%)
	Living with a partner	11 (13.9%)	6 (6.3%)	6 (7.3%)	23 (9.0%)
	Married	60 (76.0%)	84 (87.5%)	69 (84.2%)	213 (82.9%)
	Separated	4 (5.1%)	1 (1.0%)	3 (3.7%)	8 (3.1%)
	Divorced	1 (1.3%)	3 (3.1%)	1 (1.2%)	5 (2.0%)
	Don’t want to answer	0 (0%)	0 (0%)	1 (1.2%)	1 (0.4%)
Highest level of qualification	School certificate (Yr 10 or equiv)	2 (2.5%)	2 (2.1%)	0 (0%)	4 (1.6%)
	Higher school certificate (Yr 12 or equiv)	5 (6.3%)	4 (4.2%)	4 (4.9%)	13 (5.1%)
	Trade/Apprentice	7 (8.9%)	10 (10.4%)	9 (11.0%)	26 (10.1%)
	Certificate/Diploma	10 (12.7%)	11 (11.5%)	11 (13.4%)	32 (12.5%)
	University degree	30 (38.0%)	42 (43.8%)	37 (45.1%)	109 (42.4%)
	Higher University Degree	25 (31.7%)	26 (27.1%)	21 (25.6%)	72 (28.0%)
	Don’t want to answer	0 (0%)	1 (1.0%)	0 (0%)	1 (0.4%)
Employment status	Full-time paid	69 (87.3%)	89 (92.7%)	74 (90.2%)	232 (90.3%)
	Part-time paid	7 (8.9%)	4 (4.2%)	5 (6.1%)	16 (6.2%)
	Unemployed	3 (3.8%)	1 (1.0%)	2 (2.4%)	6 (2.3%)
	Don’t want to answer	0 (0%)	2 (2.1%)	1 (1.2%)	3 (1.2%)
Student status	No	72 (91.1%)	91 (94.8%)	75 (91.5%)	238 (92.6%)
	Yes, part-time student	7 (8.9%)	2 (2.1%)	6 (7.3%)	15 (5.8%)
	Yes, full-time student	0 (0%)	3 (3.1%)	1 (1.2%)	4 (1.6%)
Yearly income of household	$1 to $18,200	1 (1.3%)	0 (0%)	0 (0%)	1 (0.4%)
	$18,201 to $37,000	0 (0%)	0 (0%)	1 (1.2%)	1 (0.4%)
	$37,001 to $60,000	5 (6.3%)	3 (3.1%)	1 (1.2%)	9 (3.5%)
	$60,001 to $80,000	1 (1.3%)	3 (3.1%)	3 (3.7%)	7 (2.7%)
	$80,001 to $100,000	8 (10.1%)	6 (6.3%)	5 (6.1%)	19 (7.4%)
	$100,001 to $120,000	7 (8.9%)	7 (7.3%)	7 (8.5%)	21 (8.2%)
	$120,001 to $140,000	7 (8.9%)	13 (13.5%)	7 (8.5%)	27 (10.5%)
	$140,001 to $160,000	14 (17.7%)	10 (10.4%)	7 (8.5%)	31 (12.1%)
	$160,001 to $180,000	8 (10.1%)	12 (12.5%)	13 (15.9%)	33 (12.8%)
	$180,001 or more	25 (31.7%)	36 (37.5%)	33 (40.2%)	94 (36.6%)
	Don’t know	0 (0%)	0 (0%)	1 (1.2%)	1 (0.4%)
	Don’t want to answer	3 (3.8%)	6 (6.3%)	4 (4.9%)	13 (5.1%)
Enrolled daughters per family	One	69 (87.3%)	89 (92.7%)	72 (87.8%)	230 (89.5%)
	Two	10 (12.7%)	7 (7.3%)	9 (11.0%)	26 (10.1%)
	Three	0 (0%)	0 (0%)	1 (1.2%)	1 (0.4%)
Previous participant (before 2017)	No	73 (92.4%)	88 (91.7%)	63 (76.8%)	224 (87.2%)
	Yes	6 (7.6%)	8 (8.3%)	19 (23.2%)	33 (12.8%)

^a^Socio-economic status by population quintile for SEIFA Index of Relative Socio-economic Advantage and Disadvantage 2016

### Primary outcome—days per week meeting physical activity recommendations

Model estimates for all outcomes obtained from the mixed effects regression models along with 95% confidence intervals (95% CI) and *p*-values are shown in Table 3 (father outcomes) and Table 4 (daughter outcomes) for the full sample and with COVID-19 impacted cohorts removed. In the full sample, relative to baseline, fathers significantly increased the number of days/week meeting physical activity recommendations by 27% (95% CI = 14% to 42%, *p* < 0.001) at 10-weeks (post-program) and by 19% (95% CI = 6% to 33%, *p* < 0.001) at 12-months. This equates to ~ 0.7 day/week increase at 10-weeks and ~ 0.5 day/week increase at 12-months, when compared with baseline. Likewise, for daughters there was a significant increase by 25% at 10-weeks (95% CI = 12% to 39%, *p* < 0.001) and by 14% at 12-months (95% CI = 2% to 27%, *p* = 0.02) when compared to baseline. This equates to ~ 0.6 day/week increase at 10-weeks and ~ 0.3 day/week increase at 12-months. After removal of COVID-19 impacted cohorts, results strengthened at both time-points for fathers and at 12-months for daughters.

**Table 3:** Model estimates with 95% CI and *p*-value for father outcomes^a^ for the full sample and with COVID-19 impacted cohorts removed

*Outcome*	*Model Distribution*	*Type of Estimate*	*Sample* ^b^	10-weeks change from baseline		12-months change from baseline	
				***Estimate (95% CI)***	***P-value***	***Estimate (95% CI)***	***P-value***
***Primary outcome***							
Days/week meeting PA recommendations	Poisson	IRR	Full sample	1.27 (1.14, 1.42)	** < 0.001**	1.19 (1.06, 1.33)	** < 0.001**
			COVID-19 impacted cohorts removed	1.36 (1.19, 1.56)	** < 0.001**	1.34 (1.17, 1.54)	** < 0.001**
***Secondary outcomes***							
MVPA (mins/day)	Gaussian	Average change	Full sample	57.83 (34.85, 80.80)	** < 0.001**	44.85 (19.91, 69.78)	** < 0.001**
			COVID-19 impacted cohorts removed	74.04 (43.85, 104.24)	** < 0.001**	52.69 (21.04, 84.33)	** < 0.001**
*Co-physical activity*							
With daughter & family (days/week)	Negative Binomial	IRR	Full sample	1.50 (1.31, 1.72)	** < 0.001**	1.20 (1.04, 1.40)	** < 0.05**
			COVID-19 impacted cohorts removed	1.57 (1.32, 1.87)	** < 0.001**	1.37 (1.14, 1.65)	** < 0.001**
With daughter only (days/week)	Poisson	IRR	Full sample	2.14 (1.84, 2.49)	** < 0.001**	1.54 (1.30, 1.82)	** < 0.001**
			COVID-19 impacted cohorts removed	2.46 (2.03, 2.99)	** < 0.001**	1.68 (1.34, 2.08)	** < 0.001**
*Screen time*							
Weekday (mins/day)	Gaussian	Average change	Full sample	-26.21 (-42.48, -9.94)	** < 0.001**	37.11 (20.23, 53.99)	** < 0.001**
			COVID-19 impacted cohorts removed	-30.13 (-38.89, -21.37)	** < 0.001**	-20.51 (-29.73, -11.29)	** < 0.001**
Weekend (mins/day)	Gaussian	Average change	Full sample	-25.23 (-41.50, -8.95)	** < 0.001**	16.56 (-0.36, 33.47)	0.06
			COVID-19 impacted cohorts removed	-26.44 (-39.53, -13.34)	** < 0.001**	-19.42 (-33.19, -5.65)	** < 0.01**
*Father involvement*							
Mother support (scale range: 1 to 7)	Ordinal	OR	Full sample	3.09 (2.17, 4.39)	** < 0.001**	3.41 (2.36, 4.94)	** < 0.001**
			COVID-19 impacted cohorts removed	3.12 (2.03, 4.79)	** < 0.001**	3.58 (2.28, 5.60)	** < 0.001**
Praise and affection (scale range: 1 to 7)	Ordinal	OR	Full sample	3.64 (2.55, 5.22)	** < 0.001**	2.58 (1.79, 3.73)	** < 0.001**
			COVID-19 impacted cohorts removed	3.90 (2.52, 6.03)	** < 0.001**	2.53 (1.63, 3.94)	** < 0.001**
Time & talking together (scale range: 1 to 7)	Ordinal	OR	Full sample	6.84 (4.74, 9.86)	** < 0.001**	4.58 (3.15, 6.65)	** < 0.001**
			COVID-19 impacted cohorts removed	7.30 (4.65, 11.47)	** < 0.001**	5.07 (3.20, 8.03)	** < 0.001**
Attentiveness (scale range: 1 to 7)	Ordinal	OR	Full sample	4.18 (2.91, 5.99)	** < 0.001**	3.75 (2.58, 5.45)	** < 0.001**
			COVID-19 impacted cohorts removed	4.41 (2.84, 6.85)	** < 0.001**	3.86 (2.45, 6.08)	** < 0.001**
Family functioning (scale range: 0 to 100)	Ordinal	OR	Full sample	2.70 (1.91, 3.82)	** < 0.001**	1.89 (1.32, 2.72)	** < 0.001**
			COVID-19 impacted cohorts removed	2.73 (1.79, 4.17)	** < 0.001**	1.92 (1.24, 2.97)	** < 0.001**
*Father-daughter relationship-*							
Disciplinary warmth (scale range: 1 to 5)	Ordinal	OR	Full sample	4.46 (3.13, 6.36)	** < 0.001**	3.04 (2.12, 4.36)	** < 0.001**
			COVID-19 impacted cohorts removed	5.22 (3.39, 8.03)	** < 0.001**	3.27 (2.11, 5.07)	** < 0.001**
Personal relationships (scale range: 1 to 5)	Ordinal	OR	Full sample	5.62 (3.92, 8.04)	** < 0.001**	3.21 (2.23, 4.60)	** < 0.001**
			COVID-19 impacted cohorts removed	6.85 (4.41, 10.65)	** < 0.001**	3.57 (2.30, 5.54)	** < 0.001**

Bold denotes a significant difference

*MVPA* Moderate to Vigorous Physical Activity

^a^Adjusted for SEIFA quintile, age of father, age of eldest daughter, number of daughters enrolled in program, previous involvement in program and cohort year

^b^Full sample includes all participants in years 2017, 2018 and 2019. COVID-19 impacted cohorts removed includes participants from 2017 and 2018 only

**Table 4:** Model estimates with 95% CI and *p*-value for daughter outcomes* for the full sample and with COVID-19 impacted cohorts removed

*Outcome*	*Model Distribution*	*Type of Estimate*	*Sample* ^*b*^	10-weeks change from baseline		12-months change from baseline	
				***Estimate (95% CI)***	***P-value***	***Estimate (95% CI)***	***P-value***
***Primary outcome*** ^***a***^							
Days/week meeting PA recommendations	Poisson	IRR	Full sample	1.25 (1.12, 1.39)	** < 0.001**	1.14 (1.02, 1.27)	** < 0.05**
			COVID-19 impacted cohorts removed	1.22 (1.07, 1.39)	** < 0.01**	1.17 (1.02, 1.34)	** < 0.05**
***Secondary outcomes***							
* Screen time*							
Weekday (mins/day)	Gaussian	Average change	Full sample	-21.09 (-40.90, -1.30)	**0.04**	64.33 (43.80, 84.85)	** < 0.001**
			COVID-19 impacted cohorts removed	-18.16 (-24.93, -11.39)	** < 0.001**	-16.05 (-23.19, -8.91)	** < 0.001**
Weekend (mins/day)	Gaussian	Average change	Full sample	-29.00 (-43.51, -14.47)	** < 0.001**	8.46 (-6.66, 23.58)	0.27
			COVID-19 impacted cohorts removed	-33.48 (-45.47, -21.49)	** < 0.001**	-24.16 (-36.78, -11.54)	** < 0.001**
* Social emotional well-being*							
Total composite score (scale range: 224 to 576)	Gaussian	Average change	Full sample	30.79 (24.69, 36.89)	** < 0.001**	26.91 (20.52, 33.30)	** < 0.001**
			COVID-19 impacted cohorts removed	32.81 (25.36, 40.25)	** < 0.001**	26.70 (18.84, 34.55)	** < 0.001**
Self-awareness (scale range: 28–72)	Gaussian	Average change	Full sample	2.78 (1.81, 3.76)	** < 0.001**	2.95 (1.93, 3.97)	** < 0.001**
			COVID-19 impacted cohorts removed	2.87 (1.71, 4.03)	** < 0.001**	2.86 (1.63, 4.08)	** < 0.001**
Social awareness (scale range: 28–72)	Gaussian	Average change	Full sample	4.21 (3.13, 5.28)	** < 0.001**	3.14 (2.01, 4.26)	** < 0.001**
			COVID-19 impacted cohorts removed	4.82 (3.58, 6.07)	** < 0.001**	3.37 (2.05, 4.69)	** < 0.001**
Self-management (scale range: 28–72)	Gaussian	Average change	Full sample	5.00 (4.01, 6.00)	** < 0.001**	4.87 (3.82, 5.91)	** < 0.001**
			COVID-19 impacted cohorts removed	5.12 (3.91, 6.32)	** < 0.001**	4.58 (3.31, 5.85)	** < 0.001**
Goal-directed behaviour (scale range: 28–72)	Gaussian	Average change	Full sample	3.37 (2.47, 4.27)	** < 0.001**	2.63 (1.68, 3.57)	** < 0.001**
			COVID-19 impacted cohorts removed	3.35 (2.24, 4.47)	** < 0.001**	2.43 (1.25, 3.61)	** < 0.001**
Relationship skills (scale range: 28–72)	Gaussian	Average change	Full sample	3.54 (2.53, 4.55)	** < 0.001**	3.05 (1.99, 4.10)	** < 0.001**
			COVID-19 impacted cohorts removed	3.90 (2.69, 5.11)	** < 0.001**	3.17 (1.90, 4.45)	** < 0.001**
Personal responsibility (scale range: 28–72)	Gaussian	Average change	Full sample	4.57 (3.66, 5.47)	** < 0.001**	3.87 (2.92, 4.82)	** < 0.001**
			COVID-19 impacted cohorts removed	4.98 (3.85, 6.10)	** < 0.001**	3.94 (2.75, 5.13)	** < 0.001**
Decision making (scale range: 28–72)	Gaussian	Average change	Full sample	3.70 (2.74, 4.66)	** < 0.001**	3.36 (2.36, 4.37)	** < 0.001**
			COVID-19 impacted cohorts removed	3.90 (2.77, 5.05)	** < 0.001**	3.23 (2.03, 4.44)	** < 0.001**
Optimistic thinking (scale range: 28–72)	Gaussian	Average change	Full sample	3.77 (2.85, 4.69)	** < 0.001**	3.26 (2.30, 4.22)	** < 0.001**
			COVID-19 impacted cohorts removed	3.90 (2.81, 4.99)	** < 0.001**	3.24 (2.09, 4.39)	** < 0.001**
Self-esteem (scale range: 0 to 100)	Ordinal	OR	Full sample	4.19 (2.93, 6.01)	** < 0.001**	3.02 (2.09, 4.37)	** < 0.001**
			COVID-19 impacted cohorts removed	4.78 (3.08, 7.43)	** < 0.001**	3.08 (1.98, 4.80)	** < 0.001**

Bold denotes a significant difference

*Adjusted for SEIFA quintile, age of father, age of eldest daughter, number of daughters enrolled in program, previous involvement in program and cohort year

^a^For all outcomes, data was collected on the eldest daughter in the fathers with multiple daughters enrolled. However, for the primary outcome, data was also collected for any additional daughters enrolled in the program. Results did not change at any time point when these were added into the model for analysis

^b^Full sample includes all participants in years 2017, 2018 and 2019. COVID-19 impacted cohorts removed includes participants from 2017 and 2018 only

### Secondary outcomes

#### Fathers

All secondary outcome results for fathers can be found in Table 3. For MVPA, in the full sample, fathers, had an average increase of 57.8 min/day (95% CI = 34.9 to 80.8, *p* < 0.001) at 10-weeks and an average increase of 44.9 min/day (95% CI = 19.9 to 69.9, *p* < 0.001) at 12-months compared with baseline. After removal of COVID-19 impacted cohorts, results strengthened at both time-points.

Relative to baseline, fathers increased days/week participating in co-physical activity one-on-one with their daughter by 114% (95% CI = 84% to 149%, *p* < 0.001) at 10-weeks and by 50% (95% CI = 30% to 82%, *p* < 0.001) at 12-months when the full sample was analysed. Similarly, for days/week participating in co-physical activity with their daughter and other family members, fathers, increased days/week by 50% (95% CI = 31% to 72%, *p* < 0.001) at 10-weeks and by 20% (95% CI = 4% to 40%, *p* = 0.02) at 12-months follow-up. Results strengthened at both time-points after removal of COVID-19 impacted cohorts.

Program participation resulted in a statistically significant reduction of 26.2 min/day (95% CI = -42.5 to -9.9, *p* < 0.001) in fathers’ screen time on weekdays and a reduction of 25.2 min/day at weekends (95% CI = -41.5 to -9.0, *p* < 0.001) at 10-weeks (post-program) compared with baseline when the full sample was analysed. No significant reductions were observed at 12-months, but an inverse effect was apparent, with a significant increase in fathers’ screen time on weekdays (+ 37.1 min/day, 95% CI = 20.2 to 54.0, *p* < 0.001). An increase also occurred on weekends, but this was not significant (+ 16.6 min/day, 95% CI = -0.4 to 33.5, *p* = 0.06). However, after removal of COVID-19 impacted cohorts, there were significant reductions in screen time on weekdays at 10-weeks (-30.1 min/day, 95% CI = -38.9 to -21.4, *p* < 0.001) and 12-months (-20.5 min/day, 95% CI = -29.7 to -11.3, *p* < 0.001). Similarly, there were significant reductions in screen time on a weekend at 10-weeks (-26.4 min/day, 95% CI = -39.5 to -13.3, *p* < 0.001) and 12-months (-19.4 min/day, 95% CI = -33.2 to -5.7, *p* < 0.001).

At both 10-weeks (post-program) and 12-month follow-up there was a significant improvement in all sub-scales of father involvement when the full sample was analysed. Relative to baseline, fathers were 3.1 times more likely (95% CI = 2.2 to 4.4, *p* < 0.001) to provide ‘mother support’ (e.g., provide parenting support to mothers) at 10-weeks, and this was maintained at 12-months (OR: 3.4, 95% CI = 2.4 to 4.9, *p* < 0.001). Additionally, when compared with baseline, fathers were 3.6 times more likely (95% CI = 2.6 to 5.2, *p* < 0.001) to praise and show affection to daughters at 10-weeks, and this was sustained at 12-months respectively (OR: 2.6, 95% CI = 1.8 to 3.7, *p* < 0.001). Fathers were also 6.8 times more likely (95% CI = 4.7 to 9.9, *p* < 0.001) to spend time and talk together with daughters at 10-weeks compared with baseline, and this was maintained at 12-months, respectively (OR: 4.6, 95% CI = 3.2 to 6.7, *p* < 0.001). Finally, relative to baseline, fathers were 4.2 times more likely (95% CI = 2.9 to 6.0, *p* < 0.001) to demonstrate attentiveness with daughters at 10-weeks (post-program) and this was sustained at 12-months (OR: 3.8, 95% CI = 2.6 to 5.5, *p* < 0.001). After removal of COVID-19 impacted cohorts, results strengthened for most father involvement sub-scales at each time-point.

Compared with baseline, fathers were 2.7 times more likely (95% CI = 1.9 to 3.8, *p* < 0.001) at 10-weeks and 1.9 times more likely (95% CI = 1.3 to 2.7, *p* < 0.001) at 12-months to report an improvement in family functioning when the full sample was analysed. Results strengthened at both time-points after removal of COVID-19 impacted cohorts.

Finally, the father-daughter relationship improved as a result of program participation when the full sample was analysed. Relative to baseline, fathers were 4.5 times more likely (95% CI = 3.1 to 6.4, *p* < 0.001) post-program and 3.0 times more likely (95% CI = 2.1 to 4.4, *p* < 0.001) at 12-months to improve disciplinary warmth (e.g., praising and complementing daughter, shared decision making and providing reasoning for rules/disciplinary actions). Similarly, fathers were 5.6 times more likely (95% CI = 3.9 to 8.0, *p* < 0.001) post-program and 3.2 times more likely (95% CI = 2.2 to 4.3, *p* < 0.001) times at 12-months to improve personal relationships (e.g., demonstrating intimacy, nurturance, companionship, and prosocial behaviour). After removal of COVID-19 impacted cohorts, results strengthened for both father-daughter relationship sub-scales at each time-point.

#### Daughters

All secondary outcome results for daughters can be found in Table 4. Participation in *DADEE* resulted in a significant reduction of 21.1 min/day (95% CI = -40.9 to -1.3, *p* = 0.04) in daughters’ screen time on weekdays and a reduction of 29.0 min/day at weekends (95% CI = -43.5 to -14.5, *p* < 0.001) at 10-weeks (post-program) compared with baseline when the full sample were analysed. No significant reductions were observed at 12-months, but a significant inverse effect was apparent, with an increase in daughters’ screen time on weekdays (+ 64.3 min/day, 95% CI = 43.8 to 84.9, *p* < 0.001), while a small but non-significant effect occurred for daughters’ screen time on weekends (+ 8.5 min/day, 95% CI = -6.7 to 23.6, *p* = 0.27). However, after removal of COVID-19 impacted cohorts, there were significant reductions in screen time on weekdays at 10-weeks (-18.2 min/day, 95% CI = -24.9 to -11.4, *p* < 0.001) and 12-months (-16.1 min/day, 95% CI = -23.2 to -8.9, *p* < 0.001). Similarly, there were significant reductions in screen time on a weekend at 10-weeks (-33.5 min/day, 95% CI = -45.5 to -21.5, *p* < 0.001) and 12-months (-24.2 min/day, 95% CI = -36.8 to -11.5, *p* < 0.001).

Relative to baseline, daughters’ significantly increased total DESSA social–emotional composite score by 30.8 points (95% CI = 24.69 to 36.89, *p* < 0.001) at 10-weeks and by 26.9 points (95% CI = 20.52 to 33.30, *p* < 0.001) at 12-months when the full sample were analysed. In addition, all individual social–emotional competency scales significantly improved at 10-weeks post-program (all *p* < 0.001) and 12-month follow-up (all *p* < 0.001). After removal of COVID-19 impacted cohorts, results strengthened for total DESSA social–emotional composite score at 10-weeks but not at 12-months.

Finally, the daughters’ self-esteem improved after participating in the program when the full sample were analysed. Compared with baseline, daughters’ were 4.2 times more likely (95% CI = 2.9 to 6.0, *p* < 0.001) at 10-weeks and 3.0 times more likely (95% CI = 2.1 to 4.4, *p* < 0.001) at 12-months to have an improved self-esteem score from the 4-items of the Kindl-R questionnaire (e.g., *was proud of herself, felt on top of the world, was pleased with herself and had lots of good ideas*). After removal of COVID-19 impacted cohorts, results strengthened at both time points.

#### Process Outcomes

To determine replicability of the program, a-priori process outcome targets were set corresponding to the previous *DADEE* community RCT [14]. Findings show these targets were exceeded, as outlined below:

#### Recruitment capability (Target: 240 families to 12 DADEE programs across three years)

A total of 257 families (257 fathers + 285 daughters) were enrolled in the program and attended at least one session which exceeded the recruitment target of 240 families.

#### Attendance (Target: At least 70% attendance on average across the nine weeks)

Average attendance across all programs conducted over the three years was 80%. In addition, the average number of sessions attended was 7.2 out of 9.

#### Retention (Target of ≥ 85% of daughters and dads retained at post-program assessments)

A total of 86% (*n* = 220 families) were retained in the study at the end of the program and undertook 10-week assessments, while 78% (*n* = 201 families) were retained at 12-months follow-up and undertook 12-month assessments.

#### Program acceptability (Target: mean score of ≥ 4 out of 5 for satisfaction items measured via questionnaire using a 5-point Likert scale)

All acceptability and satisfaction findings are provided in Table 5. In summary, fathers considered the overall quality of the program and facilitators to be high. On a scale of 1 (poor) to 5 (excellent), the mean (standard deviation) for overall program satisfaction score and overall facilitators rating score (both fathers’ facilitator and daughters’ facilitator) were both 4.8 (0.4), which exceeded the *a*-priori target (≥ 4 out of 5).

**Table 5:** Acceptability and satisfaction findings as reported by fathers (*n* = 220)

Construct	Questions asked^a^	Mean	SD
Satisfaction (scale range 1 to 5)	Overall, I enjoyed the *DADEE* program	4.7	0.6
	I would recommend the program to my friends	4.8	0.4
	The benefits of the program outweighed any disruption to our normal family routine	4.6	0.5
	Overall program rating^b^	4.8	0.4
Quality of facilitators (scale range 1 to 5)	Overall rating of fathers’ facilitators^b^	4.8	0.4
	Overall rating of facilitators daughters’ facilitators^b^	4.8	0.4

^a^1 = strongly disagree; 2 = disagree; 3 = neutral; 4 = agree; 5 = strongly agree

^b^ 1 = poor; 2 = fair; 3 = average; 4 = good; 5 = excellent

## Discussion

The current study aimed to determine if the positive outcomes for fathers and daughters established in the previous *DADEE* RCTs [11, 14] could be sustained long-term (12-months) when delivered in the community by trained facilitators. Findings confirmed there were significant sustained effects on all primary and secondary outcomes in both fathers and daughters at 12-months except screen-time when the full sample were analysed. However, the follow-up periods from the 2019 programs were impacted by COVID-19 pandemic and sensitivity analysis which removed these cohorts showed a strengthening of most outcomes in fathers and daughters, in particular screentime which had significant sustained effects. Additionally, the process outcomes enhanced the external validity of these findings with all *a*-priori benchmarks met for recruitment, attendance, retention and program acceptability. Overall, results provide support for a sustained effect of the program while delivered in a community setting by trained facilitators.

Delivering community-based physical activity programs that produce effective and sustained behaviour change remains a challenge [15, 36]. However, this current study demonstrated 12-month improvements for the primary outcome (days/week meeting physical activity recommendations) in both fathers (19% increase from baseline) and daughters’ (14% increase from baseline). These findings are noteworthy considering that 68% of Australian adult males (18–64 years) do not meet the national physical activity guidelines [3], and there is usually a decline in men's physical activity during fatherhood [7]. Similarly, a decline in physical activity is observed in girls over time [37]. The difficulties in reversing this trend are evident, as indicated by the small effects from a meta-analysis of physical activity interventions among girls [38]. The importance of these significant and sustained improvements cannot be understated, especially given the paucity of family-based, physical activity programs that; target this dyad [15], are delivered in the community [39] and assess long term-impact [15].

The motivation to serve as a positive role model for their daughter and the newfound enjoyment derived from engaging in physical activities together may be a driving force for maintaining an active lifestyle amongst fathers [40]. The program likely facilitated fathers in accessing these motivational factors, as evidenced by the large rise in father-child co-physical activity observed at the 12-months. Other potential reasons for the sustained activity levels include the program’s focus on improving daughters’ FMS proficiency to enable girls to play sport confidently and competently. FMS competency provides a foundation for an active lifestyle and is strongly associated with lifelong physical activity [41]. In addition, the program provided families with the opportunity to develop the necessary psychological resources and skills to persist in practise and overcome mistakes to facilitate continuation of sport and physical activity. Finally, the program looked to combat societal gender bias that restricts girls’ participation in sport and physical activity [42] by giving daughters’ confidence and critical thinking skills and empowering fathers to be gender equity advocates for their daughters. The above explanations for physical activity maintenance align with the main theoretical themes underpinning the program to promote long-term behaviour change, which include; maintenance motives, self-regulation, physical and psychological resources and optimised social support systems [43, 44].

Prolonged improvements at 12-months were also evident for secondary outcomes for fathers MVPA, parenting practices, co-physical activity, the father-daughter relationship and daughters’ social-emotional well-being, and self-esteem. Additional reasoning behind the long-term success could be attributed to the trained facilitators who possessed the appropriate pedagogical skills to effectively deliver engaging education and practical sessions. Targeting experienced physical education teachers as facilitators also served as a ‘gateway’ to the community and local schools. This approach is more scalable than our original efficacy trial [11], which was conducted solely by the research team. Additionally, it helped with identification of suitable venues for hosting the program and recruitment of participants. The facilitators also undertook a robust 3-day training and were provided with a comprehensive support package to further enhance program delivery. Research shows that supporting individuals conducting physical activity programs for children via informal education and professional development opportunities such as training and access to resources can strengthen the execution of programs and enhance children's involvement in physical activities [45–47]. Furthermore, the program content addressed the unique values, motivators, and obstacles faced by fathers and their daughters, while also challenging gender stereotypes, norms, and unrealistic ideals for females that hinder girls' involvement in physical activity. This may have helped resonate with families, leading to greater engagement with the program.

The positive process findings for recruitment capability, attendance, retention and program acceptability provide encouragement for a sustained delivery model. However, implementation research has stressed the importance of efficiency in physical activity promotion interventions [48, 49]. Specifically, the need to optimise interventions to be delivered at scale with relatively low incremental costs [48]. This was not possible to do within the scope of the current study as funding was for a large scale roll out rather than an implementation trial. In addition. it was imperative to establish long-term impact before conducting an implementation trial. Future research on the *DADEE* program, requires a systematic evaluation of intervention components to identify the maximally efficient and effective treatment package [49]. In addition, utilising an appropriate implementation framework such as the PRACTIS guide [50] will be integral to map features of the implementation setting, identify and engage key stakeholders, and anticipate and address potential barriers and facilitators to effective implementation and scale up.

Strengths of this study include; long-term participant follow-up (12-months) with a large sample size and high retention (78% at 12-months), comprehensive process evaluation, and an intention-to-treat analysis assessing outcome effects. Despite best efforts to recruit a diverse sample, the study was limited by an over-representation of fathers with higher education levels and an under-representation of fathers who were Aboriginal and/or Torres Strait Islander origin which may limit generalisability of results. A recent scoping review highlighted the lack of Aboriginal and/or Torres Strait Islander parents as a common limitation among parenting programs for improving children’s health, and that targeted strategies are needed [51]. The study was also limited by a lack of control group and use of brief, self-report instruments to assess behaviour change. Therefore, results should be interpreted with some caution. However, these decisions were aligned with the translational nature of this trial and since efficacy and effectiveness has previously been demonstrated in two RCTs utilising extensive objective measures and a comparison group [11, 14].

## Conclusions

This study demonstrated successful long-term impact of the *DADEE* program resulting in important and sustained health benefits in both fathers and daughters over 12 months when delivered in community settings by trained facilitators. To build on this, further investigation is required to identify the most efficient implementation systems, processes and contextual factors to deliver the program at scale with relatively low incremental costs. This study adds to the growing body of research highlighting the critical role of fathers for improving the physical and social-emotional well-being of their daughters.

## Supplementary Information

Additional file 1.

Additional file 2.

## Data Availability

The datasets used and/or analysed during the current study are available from the corresponding author on reasonable request.

## Abbreviations

DADEE: Dads and Daughters Exercising and Empowered
FMS: Fundamental Movement Skills
MVPA: Moderate-to-Vigorous Physical Activity
BMI: Body Mass Index
RCT: Randomised Controlled Trial
ITT: Intention to Treat
CI: Confidence Intervals
SEIFA: Socio-Economic Indexes for Areas
