# Supplementary material for: Twelve-month outcomes of a community-based, father-daughter physical activity program delivered by trained facilitators
Source: Int J Behav Nutr Phys Act. 2024 Sep 11;21:101. doi: 10.1186/s12966-024-01648-w (PMC11389107; doi:10.1186/s12966-024-01648-w)
Supplement: Supplementary file 2 — Additional file 2. [file 12966_2024_1648_MOESM2_ESM.docx]

Additional File 2

**Twelve-month outcomes of a community-based, father-daughter physical activity program delivered by trained facilitators.**

Lee M Ashton, Anna T Rayward, Emma R. Pollock, Stevie-Lee Kennedy, Myles D. Young, Narelle Eather, Alyce T. Barnes, Daniel R Lee, Philip J. Morgan*.

***** Correspondence: Philip.morgan@newcastle.edu.au; Tel.: +61-2-49-217265

**Table of Contents**

| **Supporting information item** | **Page** |
| --- | --- |
| **Supplementary Table 1.** Number of observations, mean (SD) and median (Q1, Q3) for physical activity, screen time, parenting outcomes, daughters’ self-esteem and father-daughter relationship. | 2 |
| **Supplementary Table 2.** Number of observations, mean (SD) and median (Q1, Q3) for daughters Social-emotional wellbeing (father-proxy). | 4 |

**Supplementary Table 1.** Number of observations, mean (SD) and median (Q1, Q3) for physical activity, screen time, parenting outcomes, daughters’ self-esteem and father-daughter relationship.

|  | | ***Time point*** | | |
| --- | --- | --- | --- | --- |
| ***Variable*** | ***Category*** | ***Baseline***  ***(n = 257 Dads, n= 285 daughters)*** | ***Post Program***  ***(n =220 Dads, n=244 daughters)*** | ***12-month Follow-up (n =201 Dads, n=219 daughters)*** |
| Father MVPA (Self-reported days meeting Physical Activity recommendations- days/week) | n | 257 | 220 | 202 |
|  | mean (SD) | 2.6 (1.8) | 3.3 (1.7) | 3.1 (1.8) |
|  | median  (Q1, Q3) | 2 (1,4) | 3 (2,4) | 3 (2,4) |
| Daughter MVPA (Self-reported days meeting Physical Activity recommendations- days/week) | n | 285 | 242 | 216 |
|  | mean (SD) | 2.7 (1.6) | 3.3 (1.6) | 3.0 (1.5) |
|  | median  (Q1, Q3) | 3 (2,4) | 3 (2,4) | 3 (2,4) |
| Fathers Moderate-to vigorous physical activity (MVPA) - mins/day | n | 257 | 220 | 175 |
|  | mean (SD) | 160.2 (179.6) | 219.8 (219.3) | 200.6 (158.9) |
|  | median  (Q1, Q3) | 120 (50, 225) | 180 (120,270) | 175 (90,240) |
| Dad co-physical activity with daughter & family- days/week | n | 257 | 220 | 194 |
|  | mean (SD) | 1.4 (1.3) | 2.2 (1.5) | 1.7 (1.4) |
|  | median  (Q1, Q3) | 1 (0,2) | 2 (1,3) | 2 (1,2) |
| Dad co-physical activity with daughter only - days/week | n | 257 | 220 | 194 |
|  | mean (SD) | 1.0 (1.2) | 2.2 (1.4) | 1.5 (1.3) |
|  | median  (Q1, Q3) | 1 (0,2) | 2 (1,3) | 1 (1,2) |
| Fathers screen time – weekday mins/day | n | 257 | 219 | 192 |
|  | mean (SD) | 103.8 (63.3) | 77.0 (43.1) | 139.9 (148.5) |
|  | median  (Q1, Q3) | 96 (60, 132) | 72 (48,108) | 96 (60, 150) |
| Fathers screen time – weekend mins/day | n | 257 | 219 | 192 |
|  | mean (SD) | 133.4 (83.7) | 107.2 (62.8) | 149.0 (130.1) |
|  | median  (Q1, Q3) | 120 (75, 180) | 60 (30,112.5) | 120 (60, 180) |
| Daughters screen time weekday mins/day | n | 257 | 219 | 192 |
|  | mean (SD) | 89.1 (62.7) | 67.4 (45.2) | 153.2 (195.1) |
|  | median  (Q1, Q3) | 72 (42, 120) | 60 (30,96) | 78 (37.5, 150) |
| Daughters screen time weekend mins/day | n | 257 | 219 | 192 |
|  | mean (SD) | 147.9 (86.9) | 117.8 (60.1) | 155.0 (113.5) |
|  | median  (Q1, Q3) | 142.5 (90,180) | 120 (75,150) | 131.3 (90, 180) |
| Father involvement – Mother support – 7-point scale score (ave score from 3 Q’s). Scale range 1 to 7. | n | 257 | 218 | 190 |
|  | mean (SD) | 4.2 (1.0) | 4.6 (0.9) | 4.6 (0.9) |
|  | median  (Q1, Q3) | 4.3 (3.3, 5) | 4.7 (4, 5) | 4.7 (4, 5.3) |
| Father involvement – Praise and affection – 7-point scale score (ave score from 4 Q’s). Scale range 1 to 7. | n | 257 | 218 | 190 |
|  | mean (SD) | 4.8 (0.8) | 5.2 (0.7) | 5.1 (0.8) |
|  | median  (Q1, Q3) | 5 (4.3, 5.5) | 5.3 (4.8, 5.8) | 5.3 (4.8, 5.5) |
| Father involvement – time & talking together – 7-point scale score (ave score from 8 Q’s). ). Scale range 1 to 7. | n | 257 | 215 | 190 |
|  | mean (SD) | 3.6 (0.9) | 4.2 (0.7) | 4.1 (0.8) |
|  | median  (Q1, Q3) | 3.6 (3, 4.1) | 4.1 (3.8,4.6) | 4.1 (3.6, 4.5) |
| Father involvement – Attentiveness – 7-point scale score (ave score from 3 Q’s). ). Scale range 1 to 7. | n | 257 | 218 | 188 |
|  | mean (SD) | 4.4 (1.0) | 4.8 (0.7) | 4.8 (0.8) |
|  | median  (Q1, Q3) | 4.3 (3.7,5) | 5 (4.3, 5.3) | 5 (4.3, 5.3) |
| Daughters’ self-esteem (father proxy) – 5-point scale score. Transformed score range: 0 to 100 | n | 257 | 218 | 192 |
|  | mean (SD) | 62.6 (11.9) | 69.1 (10.8) | 67.9 (11.5) |
|  | median  (Q1, Q3) | 62.5 (50, 75) | 68.8 (62.5, 75) | 68.8 (56.3, 75) |
| Family functioning 5-point scale score. Transformed scale range: 0 to 100 | n | 257 | 218 | 193 |
|  | mean (SD) | 66.0 (14.6) | 71.2 (11.7) | 69.3 (14.1) |
|  | median  (Q1, Q3) | 68.8 (56.3, 75) | 68.8 (62.5, 75) | 68.8 (62.5, 75) |
| Father-daughter relationship- Disciplinary warmth 5-point scale score. Scale range 1 to 5. | n | 257 | 217 | 191 |
|  | mean (SD) | 3.6 (0.5) | 3.9 (0.5) | 3.8 (0.5) |
|  | median  (Q1, Q3) | 3.7 (3.3, 4) | 3.8 (3.7,4.2) | 3.8 (3.5, 4) |
| Father-daughter relationship- Personal relationships 5-point scale score. Scale range 1 to 5. | n | 257 | 217 | 191 |
|  | mean (SD) | 3.3 (0.5) | 3.6 (0.5) | 3.5 (0.5) |
|  | median  (Q1, Q3) | 3.3 (3, 3.8) | 3.6 (3.4, 4) | 3.5 (3.1, 3.9) |

**Supplementary Table 2.** Number of observations, mean (SD) and median (Q1, Q3) for daughters Social-emotional wellbeing (father-proxy).

|  | | *Time point* | | |
| --- | --- | --- | --- | --- |
| *Variable* | *Category* | *Baseline*  *(n = 257)* | *Post Program*  *(n =220)* | *12-month Follow-up (n =201)* |
| Overall composite score (scale range 224-576) | n | 257 | 218 | 192 |
|  | mean (SD) | 360.6 (56.7) | 393.3 (58.1) | 391.0 (58.2) |
|  | median  (Q1, Q3) | 362 (323,394) | 385.5 (356,429) | 390 (348,427) |
| Self-awareness (scale range 28-72) | n | 257 | 218 | 192 |
|  | mean (SD) | 44.9 (8.0) | 47.8 (8.1) | 48.2 (7.9) |
|  | median  (Q1, Q3) | 43 (41,50) | 48 (43,52) | 48 (43,52) |
| Social awareness (scale range 28-72) | n | 257 | 218 | 192 |
|  | mean (SD) | 44.6 (8.8) | 48.9 (8.7) | 48.1 (8.5) |
|  | median  (Q1, Q3) | 45 (39, 51) | 49 (43, 55) | 49 (43,53) |
| Self-Management (scale range 28-72) | n | 257 | 218 | 192 |
|  | mean (SD) | 45.1 (8.1) | 50.4 (8.7) | 50.3 (8.7) |
|  | median  (Q1, Q3) | 44 (39, 51) | 50 (44,57) | 51 (44,56) |
| Goal directed behaviour (scale range 28-72) | n | 257 | 218 | 192 |
|  | mean (SD) | 46.4 (8.5) | 50.0 (7.7) | 49.6 (7.9) |
|  | median  (Q1, Q3) | 47 (41,50) | 50 (44, 55) | 49 (44, 55) |
| Relationship Skills (scale range 28-72) | n | 257 | 218 | 192 |
|  | mean (SD) | 44.8 (9.0) | 48.5 (9.1) | 48.2 (9.6) |
|  | median  (Q1, Q3) | 46 (40, 50) | 48 (44,54) | 48 (43, 52) |
| Personal responsibility (scale range 28-72) | n | 257 | 218 | 192 |
|  | mean (SD) | 44.8 (8.3) | 49.5 (8.2) | 49.1 (8.3) |
|  | median  (Q1, Q3) | 44 (40, 50) | 50 (44, 55) | 50 (44, 53) |
| Decision making (scale range 28-72) | n | 257 | 218 | 192 |
|  | mean (SD) | 45.7 (8.2) | 49.6 (8.7) | 49.5 (8.7) |
|  | median  (Q1, Q3) | 47 (40,52) | 49 (45, 54) | 49 (45,54) |
| Optimistic thinking (scale range 28-72) | n | 257 | 218 | 192 |
|  | mean (SD) | 44.3 (8.5) | 48.4 (8.3) | 48.1 (8.2) |
|  | median  (Q1, Q3) | 43 (39, 49) | 47 (43, 52) | 47 (43, 52) |
